# Conditions for the stable adsorption of lipid monolayers to solid surfaces

**DOI:** 10.1093/pnasnexus/pgad190

**Published:** 2023-06-07

**Authors:** Marin Šako, Fabio Staniscia, Emanuel Schneck, Roland R Netz, Matej Kanduč

**Affiliations:** Department of Theoretical Physics, Jožef Stefan Institute, Jamova 39, Ljubljana, 1000, Slovenia; Department of Theoretical Physics, Jožef Stefan Institute, Jamova 39, Ljubljana, 1000, Slovenia; Department of Physics, Technische Universität Darmstadt, Hochschulstrasse 8, Darmstadt 64289, Hesse, Germany; Fachbereich Physik, Freie Universität Berlin, Berlin 14195, Germany; Department of Theoretical Physics, Jožef Stefan Institute, Jamova 39, Ljubljana, 1000, Slovenia

**Keywords:** lipid layers, contact angle, surface tension, wetting, molecular dynamics simulation

## Abstract

Lipid monolayers are ubiquitous in biological systems and have multiple roles in biotechnological applications, such as lipid coatings that enhance colloidal stability or prevent surface fouling. Despite the great technological importance of surface-adsorbed lipid monolayers, the connection between their formation and the chemical characteristics of the underlying surfaces has remained poorly understood. Here, we elucidate the conditions required for stable lipid monolayers nonspecifically adsorbed on solid surfaces in aqueous solutions and water/alcohol mixtures. We use a framework that combines the general thermodynamic principles of monolayer adsorption with fully atomistic molecular dynamics simulations. We find that, very universally, the chief descriptor of adsorption free energy is the wetting contact angle of the solvent on the surface. It turns out that monolayers can form and remain thermodynamically stable only on substrates with contact angles above the *adsorption contact angle*, $\theta_{\text{ads}}$. Our analysis establishes that $\theta_{\text{ads}}$ falls into a narrow range of around 60${}^{\circ }$–70${}^{\circ }$ in aqueous media and is only weakly dependent on the surface chemistry. Moreover, to a good approximation, $\theta_{\text{ads}}$ is roughly determined by the ratio between the surface tensions of hydrocarbons and the solvent. Adding small amounts of alcohol to the aqueous medium lowers $\theta_{\text{ads}}$ and thereby facilitates monolayer formation on hydrophilic solid surfaces. At the same time, alcohol addition weakens the adsorption strength on hydrophobic surfaces and results in a slowdown of the adsorption kinetics, which can be useful for the preparation of defect-free monolayers.

**Significance Statement**

Lipids can spontaneously self-assemble into bilayer structures and adsorb to various interfaces as dense monolayers. Such monolayers are essential in our lungs and eyes and are used in technological applications, for instance, to increase biocompatibility and block undesirable adhesion. Experience tells us that a monolayer will only form if the surface is hydrophobic enough. However, a precise quantitative “hydrophobicity threshold” for monolayer adsorption has never been formulated. We demonstrate that the surface contact angle is the decisive parameter controlling monolayer formation, nearly irrespective of other chemical details. Stable monolayers can form only on surfaces whose contact angles exceed a threshold of 65$\pm 5^{\circ }$. This universal insight can serve as a guiding principle for applications utilizing lipid monolayers as surface coatings.

## Introduction

Lipids, an abundant component of all biological matter, have the remarkable ability to spontaneously self-organize into various supramolecular assemblies (1). The characteristic feature of these aggregates is the well-known bilayer structure in which two monolayers of lipids point their hydrophobic tails to each other. In contrast to lipid bilayers, lipid monolayers can also assemble at interfaces between the aqueous medium and a nonpolar, hydrophobic medium, where they drastically reduce the interfacial tension with respect to the bare interface (2). Lipid layers, either in the form of solid-supported monolayers (3, 4) or bilayers (5–7) or as monolayers at air–water interfaces (Langmuir monolayers) (8–10), offer a versatile platform for fundamental studies in the fields of membrane biophysics, nanotechnology, and biochemistry. Lipid layers are also involved in a multitude of applications, ranging from antiadhesive, antimicrobial, and antifouling surface coatings (11–14) to biosensors (15) and controlled drug delivery by lipid nanoparticles (16–18). In the latter, very intensive research field, coating nanoparticle surfaces with lipids emerged as a crucial step to ensure their colloidal stability and biocompatibility. Nature uses this concept in the case of lipoproteins—the major transporters of fats and cholesterol in the human body (19). Lipid layers have also been suggested to enhance the biocompatibility of implant surfaces against adverse immunological reactions, blood coagulation, biodegradation, protein adsorption, and adhesion of cells and bacteria (20–22). In lipid-based monolayer coatings, the lipid tails point to the hydrophobic phase, and the hydrophilic headgroups to the aqueous phase. In doing so, they link a water-incompatible hydrophobic structure with the aqueous environment. It has long been empirically established that a hydrophobic surface is a prerequisite for lipid monolayers to form (1, 3, 4, 16, 23, 24, 25, 26). In contrast, on hydrophilic surfaces, only bilayers can form under certain conditions (1, 25, 26, 27, 28, 29). The prime descriptor of surface hydrophobicity is the water contact angle. However, remarkably few studies have considered the relation between the formation of lipid monolayers on a solid surface with the contact angle of the surface (25). A quantitative link between the formation/adsorption of lipid monolayers and the contact angle of the solid surface has therefore still been lacking.

Here, we present a basic thermodynamic analysis that relates the water contact angle of the solid surface to the formation of lipid monolayers. Our central question concerns the minimum contact angle required for the stable adsorption of a lipid monolayer onto a solid surface. Indeed, we obtain a threshold value for which we introduce the term *adsorption contact angle*, $\theta_{\text{ads}}$, above which monolayer adsorption occurs. We corroborate our reasoning with atomistic molecular dynamics simulations, which provide insights into the molecular interactions associated with the adsorption. We first focus on a pure aqueous medium and, after that, also show how gradually exchanging the solvent with an organic component affects monolayer adsorption.

## Results and discussion

### Monolayer adsorption in water

To resolve the basic conditions for lipid monolayer formation, we introduce a simple system of a solid substrate in an aqueous environment containing nonionic lipids. The substrate’s surface, which is assumed to be electroneutral, nonpolarizable, and molecularly flat, is characterized by the water contact angle θ. We suppose that the total concentration of lipids is well above their critical micelle concentration (CMC) so that they form bilayer aggregates in bulk, such as vesicles and bilayer disks, and monolayers on surfaces. Individual free lipids in bulk and lipids individually adsorbed to surfaces can be safely neglected because of the extremely low CMCs of all relevant lipids. The question then is, what kind of aggregates will form on the substrate in thermal equilibrium? We can envisage three distinct scenarios: (i) no adsorption, (ii) a weakly adsorbed bilayer, or (iii) an adsorbed monolayer. In such a simple setting, bilayers can nonspecifically adsorb only through attractive van der Waals forces, which are much weaker than hydrophobic interactions involved in monolayer adsorption, as we will comment on afterward. Therefore, a bilayer in bulk or weakly adsorbed to the surface (scenarios i and ii) will not be distinguished.

**Fig. 1.**
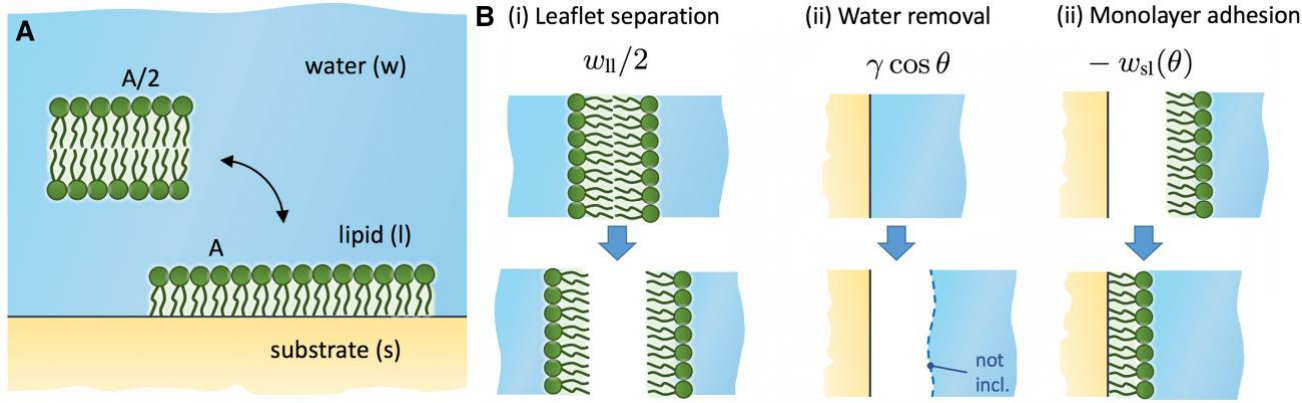
(A) Schematic depiction of an unbound bilayer of surface area A/2 and an adsorbed monolayer of surface area *A* to a solid substrate with the contact angle θ. (B) Contributions in Eq. 1 of the hypothetical thermodynamic route for transforming a bilayer into an adsorbed monolayer: (i) The bilayer leaflets are separated across vacuum, (ii) the water is removed from the substrate (no water–vapor interface), and (iii) the lipid monolayer is attached to the dry substrate.

This brings us to a two-state model of a lipid layer that can exist either as an adsorbed monolayer of surface area *A* or as a bilayer of surface area A/2, as depicted in Fig. 1A. The question of whether a monolayer can form in thermodynamic equilibrium is then equivalent to the question of which of the two states has a lower free energy. Based on the general principles of water-mediated interactions between flat surfaces (30, 31), the free energy difference between the adsorbed monolayer and the bilayer states, referred to as the *adsorption free energy*, can be calculated from the following hypothetical three-stage thermodynamic route, depicted in Fig. 1B.

In the first step (i), we separate both leaflets of the bilayer across vacuum, which requires the work $(A/2)w_{\mathrm{ll}}$. With $w_{\mathrm{ll}}$ we denoted the lipid–lipid (ll) monolayer adhesion tension in vacuum. Next, we have to remove the water adsorbed on the substrate region of area *A* onto which the lipid monolayer will adsorb (step (ii) in Fig. 1B). The free energy cost for this water removal is $A(\gamma_{\mathrm{sv}}-\gamma_{\mathrm{sw}})=A\gamma \cos\;\theta$, where $\gamma_{\mathrm{sv}}$ is the substrate–vapor and $\gamma_{\mathrm{sw}}$ substrate–water surface tension. We have used the Young equation to express their difference in terms of the *water adhesion tension*, γcosθ, where θ is the water contact angle on the substrate and γ the water–vapor surface tension. Finally, we attach the monolayer to the dry substrate (step (iii)) and gain the free energy of $-Aw_{\mathrm{sl}}$, where $w_{\mathrm{sl}}$ is the substrate–lipid (sl) monolayer adhesion tension across vacuum. In general, this term is contact angle dependent, $w_{\mathrm{sl}}(\theta )$. Note that in total, no water–vapor interface is created or eliminated in the process, therefore, we disregarded possible temporary water–vapor interfaces that could accompany the above steps. Summing up all three contributions of the described route yields the adsorption free energy per monolayer area


(1) $$\Delta F/A=w_{\mathrm{ll}}/2-w_{\mathrm{sl}}(\theta )+\gamma \cos\;\theta .$$

This equation is the basis of our forthcoming analysis. Unlike the third term, the first two are generally not easily accessible experimentally, therefore, we shall rely on computer modeling. While the lipid–lipid work of adhesion ($w_{\mathrm{ll}}$) only depends on the lipid type, the substrate–lipid work of adhesion ($w_{\mathrm{sl}}$) obviously depends also on the substrate. However, despite chemical specificity, it will turn out that $w_{\mathrm{sl}}(\theta )$ is only very weakly dependent on the substrate materials involved.

**Fig. 2.**
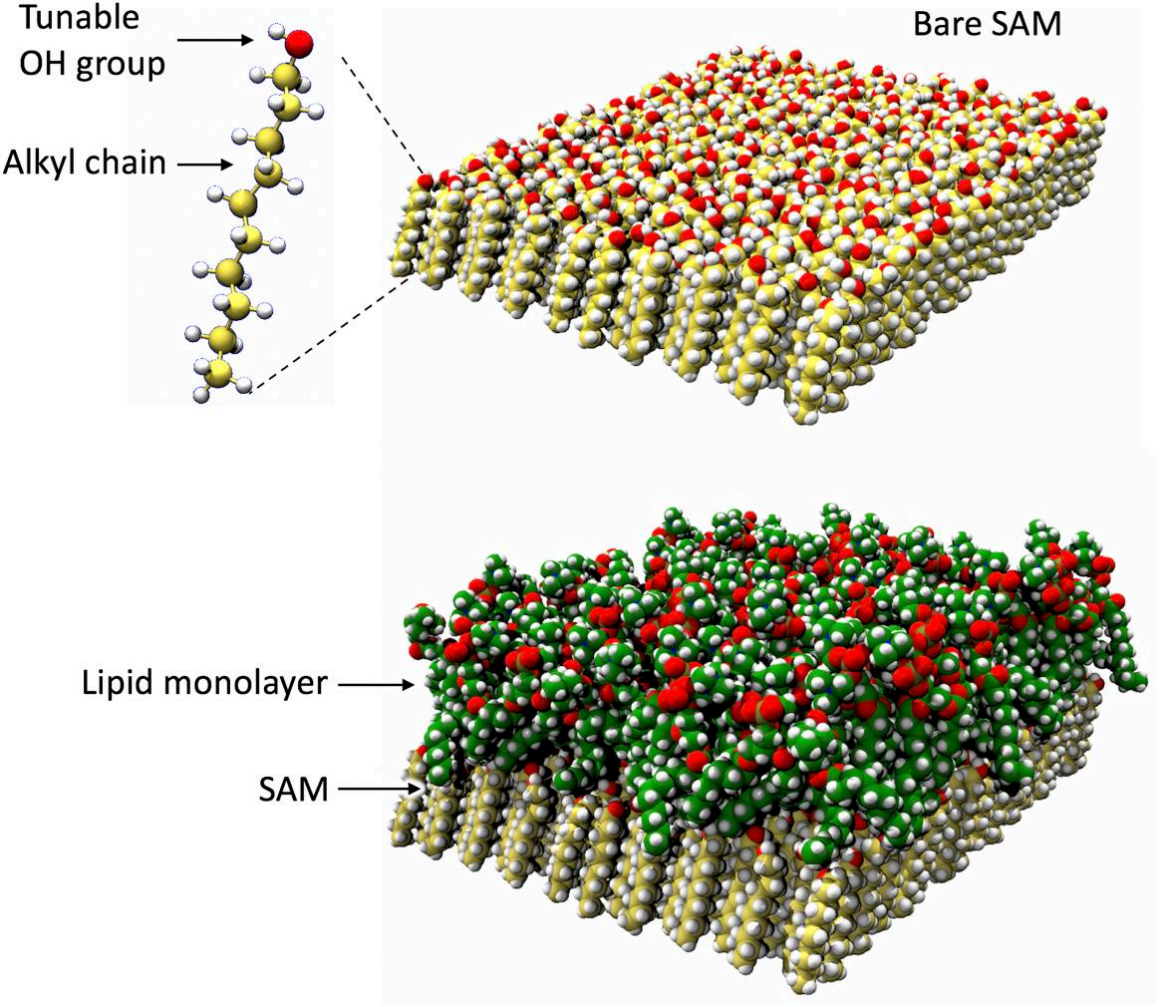
Snapshots of the MD model. Top: Bare SAM substrate, composed of hexagonally arranged alkyl molecules with OH termini with tunable polarity. Bottom: A lipid monolayer adsorbed on the SAM. The snapshots correspond to 2×2 replications of the simulation box in lateral directions.

To gain the essential principles behind the terms $w_{\mathrm{ll}}$ and $w_{\mathrm{sl}}$, we turn to classical fully atomistic molecular dynamics (MD) simulations of lipid layers and a solid substrate. These simulations account for pairwise additive interaction forces between atoms arising from Lennard-Jones and Coulomb potentials representing the van der Waals and electrostatic interactions between atomic partial charges, respectively (32, 33). The substrate is modeled as a self-assembled monolayer (SAM) of alkyl chains terminated by modified hydroxyl (OH) groups, as shown in Fig. 2. By tuning the partial charges on the O and H atoms, and thereby the OH dipoles, we cover the whole spectrum of contact angles, ranging from $\theta =0^{\circ }$ for the original hydroxyl dipole strength, up to $\theta =113^{\circ }$ for completely nonpolar OH groups, which is close to general atomically flat nonpolar surfaces (34). The choice of the lipid type in the simulations is of lesser importance, as only the alkyl tails govern the interactions in question, whereas the head groups are not directly involved. For simplicity, we choose dilauroyl-phosphatidylcholine (DLPC), which has alkyl tails composed of 12 carbon atoms. More details on the MD simulations are given in the Methods section.

The adhesion free energies are determined by integrating the pressure–distance curves obtained by summing over the perpendicular components of all interaction forces while reversibly separating both leaflets of a bilayer (for $w_{\mathrm{ll}}$) or an adsorbed monolayer from the substrate (for $w_{\mathrm{sl}}$). The details are described in the Supplementary material (see Figs. S4 and S5). We assume the same area per lipid in the adsorbed monolayer as in the bilayer. Possible small variations in the area per lipid do not cause any significant differences in adhesion owing to the liquid-like behavior of lipid tails, as we show in the Supplementary material (Table S2 and Fig. S7). In this way, we obtain the work of adhesion $w_{\mathrm{ll}}=49(1)$ mN/m for separating two DLPC leaflets. This value is roughly twice the value of the alkane–vapor surface tension, being 23–28 mN/m (35). This is hardly surprising as separating two monolayer leaflets essentially creates two alkane surfaces. The simulations in which we separate the monolayer from the substrate give the substrate–lipid work of adhesion that increases nearly linearly from $w_{\mathrm{sl}}=52(1)$ mN/m for the nonpolar substrate (with $\theta =113^{\circ }$) to 57(1) mN/m for the most polar one (with $\theta =0^{\circ }$). The prevalent interactions between the substrate and the lipids are dispersion interactions, like in the case of two lipid monolayers, therefore, $w_{\mathrm{sl}}$ is quite similar in size to $w_{\mathrm{ll}}$. The observed small variation of 10% across the entire polarity range stems from a weak electrostatic interaction of OH dipoles of the SAM with slightly polar alkyl tails of the lipids (33). This contact angle dependence in $w_{\mathrm{sl}}(\theta )$ presumably is model-specific. To analyze this effect adequately, one would need to use a chemically more accurate substrate model (e.g. with a mix of polar OH and nonpolar CH${}_{3}$ terminals (36)), which is left for future work.

**Fig. 3.**
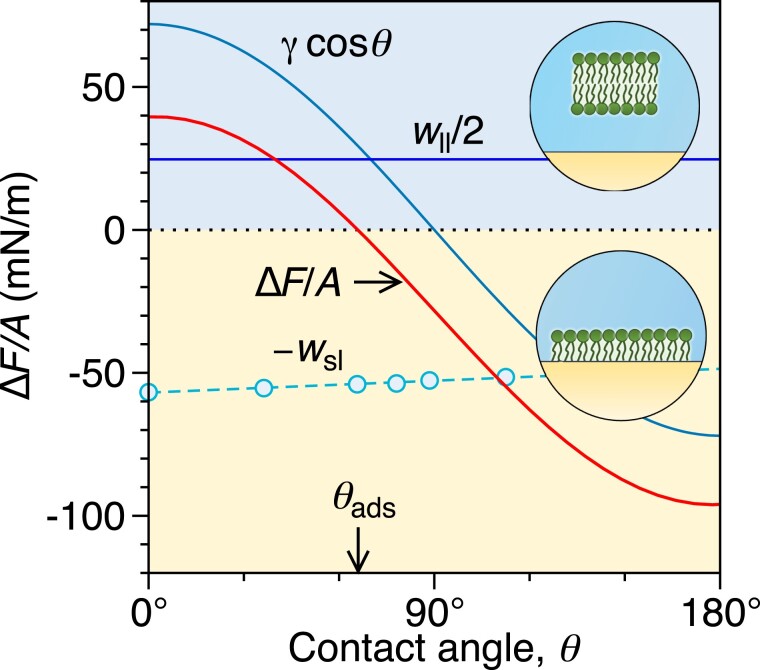
Monolayer adsorption free energy Δ*F*/*A* in water as a function of the substrate contact angle resulting from Eq. 1, along with its three contributions, namely the bilayer separation work of adhesion, $w_{\mathrm{ll}}$, the monolayer–substrate work of adhesion, $w_{\mathrm{sl}}$, and the water adhesion, γcosθ. The blue circles are explicit MD evaluations of $w_{\mathrm{sl}}$, which are fitted by a linear function (dashed line). The monolayer adsorption free energy turns from positive to negative at $\theta_{\mathrm{ads}}=66^{\circ }$.

All three contributions of Eq. 1 are shown as a function of θ in Fig. 3 by blue-shaded lines. We use γ=72 mN/m (37) for calculating the water adhesion tension, γcosθ. The MD data for $w_{\mathrm{sl}}$ (blue circles) are fitted by a linear function (dashed line). Summing up all three terms gives the monolayer adsorption free energy (Eq. 1), shown as a red solid line. The monolayer adsorption free energies are tens of mN/m in magnitude, which is much larger than the adsorption free energies of a bilayer, being below ∼1 mN/m (estimated from the van der Waals attraction in the Supplementary material). This justifies our treatment, in which we do not distinguish between an adsorbed and a nonadsorbed bilayer. The monolayer adsorption free energy is positive (ΔF>0) for small contact angles (hydrophilic surfaces), favoring the bilayer over the monolayer. In these cases, a monolayer does not form, and adding more lipids to the system will only result in more bilayer aggregates. With an increasing contact angle, ΔF monotonically decreases and becomes negative (ΔF<0) for less hydrophilic, that is, more hydrophobic surfaces, in which case the adsorbed monolayer is favored over the bilayer. In the latter scenario, lipids cover the substrate in the form of a monolayer before they start forming bilayer aggregates in bulk. They do so until the whole substrate is coated by a monolayer.

The two-state model establishes a well-defined threshold, termed the *adsorption contact angle*$\theta_{\mathrm{ads}}$, above which adsorbed monolayers are thermodynamically stable against transformation into bilayers. The adsorption contact angle is defined by the condition $\Delta F(\theta_{\mathrm{ads}})=0$, from which the expression for the adsorption contact angle follows as


(2) $$\cos\;\theta_{\mathrm{ads}}=\frac{2w_{\mathrm{sl}}(\theta_{\mathrm{ads}})-w_{\mathrm{ll}}}{2\gamma }.$$

Using the results of our MD model gives $\theta_{\mathrm{ads}}=66^{\circ }$. This value lies in the hydrophilic regime, where $\theta <90^{\circ }$. Thus, monolayers can form on weakly hydrophilic and hydrophobic surfaces, whereas very hydrophilic surfaces resist monolayer formation.

The above evaluation inevitably poses the question of how universal the value of $66^{\circ }$ is and to what extent it depends on the substrate type. The only quantity that depends on the substrate type in Eq. 2 is $w_{\mathrm{sl}}$, which is primarily governed by dispersion interactions between the substrate atoms and lipid tails. Consequently, we expect that $w_{\mathrm{sl}}$ scales linearly with the density of substrate atoms of a given type. Solid polymer materials in terms of their densities are similar to oils (0.7–0.9 g/cm${}^{3}$) (38, 39) and lipid hydrocarbon chains. A 20% variation in densities and in $w_{\mathrm{sl}}$ over a wide range of organic matter gives a $10^{\circ }$ variation in $\theta_{\mathrm{ads}}$ based on Eq. 2.

An alternative estimate, more generally applicable to solid surfaces of all sorts, can be obtained in the following way: Lipid tails can be treated approximately as a hydrocarbon liquid. The work for separating two leaflets is then approximately the same as the work needed to create two hydrocarbon (hc) interfaces, thus, $w_{\mathrm{ll}}\approx 2\gamma_{\mathrm{hc}}$, where $\gamma_{\mathrm{hc}}$ stands for the hydrocarbon–vapor surface tension. Furthermore, the work for separating a hydrocarbon liquid from a solid surface is $w_{\mathrm{sl}}=\gamma_{\mathrm{hc}}(1+\cos\;\theta_{\mathrm{hc}})$, where $\theta_{\mathrm{hc}}$ is the contact angle of liquid hydrocarbons on that solid surface. With these approximative expressions for $w_{\mathrm{ll}}$ and $w_{\mathrm{sl}}$, Eq. 2 becomes


(3) $$\cos\;\theta_{\mathrm{ads}}\approx \frac{\gamma_{\mathrm{hc}}}{\gamma }\cos\;\theta_{\mathrm{hc}}.$$

Because of their low surface tension, alkanes generally wet most solid surfaces very well, including silica (40) and graphite (41), such that $\theta_{\mathrm{hc}}$ is very small or even zero. Hence, to a good approximation, we may assume $\cos\;\theta_{\mathrm{hc}}\approx 1$, and with that, Eq. 3 further reduces to


(4) $$\cos\;\theta_{\mathrm{ads}}\approx \frac{\gamma_{\mathrm{hc}}}{\gamma }.$$

For alkane liquids ranging from C10 to C18, the surface tension spans in the range of $\gamma_{\mathrm{hc}}=23-28$ mN/m (35), for which Eq. 4 predicts $\theta_{\mathrm{ads}}\approx 67^{\circ }-71^{\circ }$. The above estimates lead to an important conclusion that the adsorption contact angle $\theta_{\mathrm{ads}}$ is quite universal in aqueous environments and almost independent of the chemical details of the surface. These conclusions may however be of limited applicability to highly charged or highly polarizable surfaces (e.g. metals) because in these cases additional interactions may further promote or suppress adhesion.

A thorough experimental examination of the adsorption contact angle has never been done. We believe that the transition between adsorbed and nonadsorbed states is subject to considerable kinetic barriers and prone to hystereses, making precise analysis challenging. Lenz et al. (25) reported that bilayers formed on substrates with $\theta <61^{\circ }$ and monolayers on substrates with $\theta >109^{\circ }$, suggesting that $\theta_{\mathrm{ads}}$ lies somewhere between $61^{\circ }$ and $109^{\circ }$, in agreement with our predictions.

As we have seen from Fig. 3, $w_{\mathrm{sl}}(\theta )$ depends only weakly on the contact angle, which allows deducing another practical approximation for the adhesion free energy. In a fairly good approximation, we can replace the function $w_{\mathrm{sl}}(\theta )$ in Eq. 1 by the value $w_{\mathrm{sl}}(\theta_{\mathrm{ads}})$. This simplification then allows expressing the first two terms in Eq. 1 by $\cos\;\theta_{\mathrm{ads}}$ using Eq. 2, which leads to a simple expression for the monolayer adsorption free energy


(5) $$\Delta F/A\approx \gamma (\cos\;\theta -\cos\;\theta_{\mathrm{ads}}).$$

The equation implies that the monolayer adsorption free energy is a linear function of cosθ around $\cos\;\theta_{\mathrm{ads}}$. Moreover, since γ depends on the solvent, and $\theta_{\mathrm{ads}}$ is quite universal for a wide range of materials, the adsorption strength for monolayer adsorption depends solely on the contact angle of the substrate. This is an important conclusion, highlighting the significance of the contact angle as the exclusive parameter that controls the substrate propensity for lipid monolayer adsorption.

Our analysis built on Eq. 1 is valid for sufficiently large lipid layers. For smaller layers, additional contributions can come to the fore, such as edge tensions of the monolayer and bilayer (in the case of a bilayer disk) and bending energy (in the case of a bilayer vesicle). These finite-size corrections are assessed quantitatively in the Supplementary material (Fig. S8), with the conclusion that they only become important for monolayer patches with radii smaller than about 10 nm.

### Monolayer adsorption at the air–water interface

A special case of a substrate is the air–water interface, the most investigated interface for lipid adsorption, particularly for creating Langmuir monolayers. Langmuir monolayers of insoluble amphiphiles and biomolecules remain a lively area of interest for modern science and technology with plenty of potential applications in hi-tech industries (1). Note that the considerations about the universality of $\theta_{\mathrm{ads}}$ for solid substrates (Eq. 4) do not apply to the air–water interface. However, Eq. 1 is general and valid also for the air–water interface by setting $w_{\mathrm{sl}}=0$, as the interaction with air is negligible, and $\theta =180^{\circ }$, as the air phase represents a perfect hydrophobic interface. The monolayer adsorption free energy to the air–water interface is then


(6) $$\Delta F_{\mathrm{air}}/A=w_{\mathrm{ll}}/2-\gamma$$

which results in $\Delta F_{\mathrm{air}}/A=-48$ mN/m based on our simulation data for $w_{\mathrm{ll}}$. The negative value implies that a full monolayer at the air–water interface is preferable over the bilayer in the bulk.

It is instructive to calculate the *equivalent contact angle*$\theta^{*}$ of a solid substrate that has the same adsorption free energy as the air–water interface. We do this by equating Eqs. 1 and 6, which brings us to the expression $\cos\;\theta^{*}=w_{\mathrm{sl}}(\theta^{*})/\gamma -1$. From the data for our system, we compute the equivalent contact angle $\theta^{*}\approx 107^{\circ }$. According to this analysis, solid surfaces with contact angles $\theta >\theta^{*}$ are stronger adsorbers of monolayers than the air–water interface. This suggests that in thermal equilibrium, monolayers will form on these surfaces at lower lipid concentrations than needed for creating a full Langmuir layer at the air–water interface. However, the extremely slow exchange rate of lipids between the interfaces and the bulk may make any experimental observation difficult in practice.

### Solvent exchange

A practical way of controlling the monolayer adsorption affinity is by tuning the property of the solvent. This can be achieved by adding a water-miscible organic solvent to water (typically alcohol), which is a more accessible approach than modifying the substrate functionalization. For instance, in the solvent-exchange method for lipid deposition, one starts from dissolved lipids in an organic solvent and then gradually replaces it with water, causing the lipids to deposit on the substrate (42–44). Our thermodynamic model for lipid coating formation (Eq. 1), so far demonstrated for an aqueous environment, also applies to other solvents that do not dissolve lipids and retain the integrity of the monolayer and bilayer. In what follows, we elucidate the thermodynamics of monolayer adsorption when water is gradually replaced by another solvent, which we showcase for the ethanol/water mixture.

The solvent generally affects all three terms in Eq. 1, although the first two only indirectly by affecting the monolayer and bilayer structures. Specifically, adding short-chained alcohols to water increases the lateral area per lipid and decreases the bilayer thickness, but the volume remains approximately constant (45, 46). Consequently, both works of adhesion, $w_{\mathrm{ll}}$ and $w_{\mathrm{sl}}$, which depend on the density of carbon atoms, should remain largely unaffected. We confirm the latter assumption with computer simulations in which we emulate the effect of ethanol by laterally expanding the lipid monolayer and bilayer, as described in detail in the Supplementary material (Figs. S6 and S7 and Tables S2 and S3). Even though we do observe small changes in both adhesion terms, they can be readily neglected for purposes of identifying the leading-order effects of solvent exchange. Note that although the area per lipid increases by up to 20% in alcohol/water solutions (45, 46), this does not affect free energies when expressed per surface area, as the absolute area drops out from the equations. Introducing ethanol into water (below the lipid solubility limit) thus influences almost exclusively the third term, γcosθ, in Eq. 1.

**Fig. 4.**
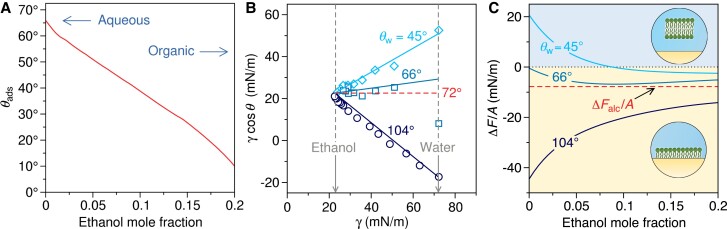
(A) Adsorption contact angle in an ethanol/water mixture as a function of ethanol mole fraction calculated from Eq. 2 by using γ from fitted experimental data. (B) Liquid adhesion tension versus air–liquid surface tension for three different substrates from literature: oxidized crystalline silicon (with water contact angles $\theta_{w}\approx 45^{\circ }$) (47), unoxidized crystalline silicon ($\theta_{w}\approx 66^{\circ }$) (47), and silanized glass ($\theta_{w}\approx 104^{\circ }$) (37). Solid lines are the predictions of Eq. 8. The data point for $\theta_{w}\approx 66^{\circ }$ in neat water is off the trend and was excluded from the fit (see Fig. S9). The dashed line shows the limiting scenario for the critical water contact angle $\theta_{w}^{c}=72^{\circ }$, defined as $\cos\;\theta_{w}^{c}=\gamma_{\mathrm{alc}}/\gamma_{w}$, for which the adhesion tension of the mixture is independent of the composition. (C) Monolayer adsorption free energy in ethanol/water solution as a function of ethanol mole fraction based on Eq. 1 for the three systems in B. The dashed line shows the hypothetical limit of the model in neat ethanol.

It is interesting first to look at how the adsorption contact angle $\theta_{\mathrm{ads}}$ changes as we move from a pure water environment to one with a higher organic content. The solvent mainly affects $\theta_{\mathrm{ads}}$ through the air–liquid surface tension γ in the denominator of Eq. 2 while the numerator ($2w_{\mathrm{sl}}(\theta_{\mathrm{ads}})-w_{\mathrm{ll}}$) remains mainly unaffected, as we have clarified above. Thus, the adsorption contact angle depends primarily on the solvent and much less so on the substrate. The surface tension of ethanol/water mixtures ranges from $\gamma_{w}=72$ mN/m in neat water down to around $\gamma_{\mathrm{alc}}=22$ mN/m in neat ethanol (37) (in this section, we will use the subscripts “w” and “alc” to refer to quantities in neat water and neat alcohol, respectively). Figure 4 A shows $\theta_{\mathrm{ads}}$ calculated from Eq. 2 where we have used γ fitted to experimental data (37) (see Fig. S9). We witness approximately a linear decrease of $\theta_{\mathrm{ads}}$ with ethanol mole fraction, from $66^{\circ }$ in neat water down to $\approx 10^{\circ }$ in a 20 mol% ethanol solution. Thus, the less polar the solvent, the more “solvophilic” the substrate should be to remain resistant to monolayer formation.

Note, however, that the addition of such organic cosolvents generally shifts also contact angles θ to lower values, which may compensate or even over-compensate for the decrease in $\theta_{\mathrm{ads}}$ for a given surface type. Hence, the effect of cosolvent on the monolayer adsorption is more complicated than just examining $\theta_{\mathrm{ads}}$. Namely, while γ decreases with alcohol concentration (37), cosθ increases at the same time, as surfactants enhance wetting quite universally (48, 49). Obviously then, the two antagonistic effects of alcohol partially cancel in the product γcosθ, which is not universal but can either increase or decrease, depending on the substrate type. In order to demonstrate the effect of solvent exchange on the adsorption free energy as dictated by Eq. 1, we resort to published experimental data (47, 37) to evaluate γcosθ for a few examples. These include measured contact angles of ethanol/water solutions on three different surfaces: two types of crystalline silicon with water contact angles $\theta_{w}\approx 45^{\circ }$ (with oxide coating) and $\theta_{w}\approx 66^{\circ }$ (unoxidized) (47) and a silanized glass with $\theta_{w}=104^{\circ }$ (37). We fit the measured cosθ and γ (37) data as a function of ethanol mole fraction (see Fig. S9), which enables us to calculate the product γcosθ for an arbitrary ethanol mole fraction. It is illuminating to plot the liquid adhesion tension for the three different substrates against γ, which we do in Fig. 4B (shown by symbols). The trend depends on the water contact angle, but all three cases almost linearly converge into a single point in neat ethanol.

The observed trends can be understood in terms of the well-established Zisman plot—an empirical relation between the contact angles and the surface tensions of various liquids on a given substrate. The revised and improved version of the Zisman plot by Bera et al. (50) suggests a linear relationship between cosθ and 1/γ of a probe liquid. Multiplying the relation by γ brings us to the following ansatz for the liquid adhesion tension


(7) $$\gamma \cos\;\theta =c_{0}+c_{1}\gamma ,$$

where $c_{0}$ and $c_{1}$ are parameters, which depend on the substrate type, and should be determined by two reference states. The first reference state is neat water, for which the surface tension ($\gamma_{w}$) and contact angle ($\theta_{w}$) are known. The other reference state is neat ethanol, which, owing to its hydrophobicity, wets most surfaces reasonably well, with $\cos\;\theta_{\mathrm{alc}}$ approaching unity, such that we may assume $\gamma \cos\;\theta \approx \gamma_{\mathrm{alc}}$ for neat ethanol. With these two boundary conditions, Eq. 7 becomes


(8) $$\gamma \cos\;\theta =\gamma_{\mathrm{alc}}+(\gamma -\gamma_{\mathrm{alc}})\;\frac{\gamma_{w}\cos\;\theta_{w}-\gamma_{\mathrm{alc}}}{\gamma_{w}-\gamma_{\mathrm{alc}}},$$

where the surface tension γ is the only parameter that characterizes the ethanol/water mixture.

The predictions of Eq. 8 are plotted in Fig. 4B as solid lines; they agree very well with the experimental data points (apart from one outlier point in neat water). The slope is dictated by the water contact angle and vanishes at the critical value of $\theta_{w}^{c}\approx 72^{\circ }$, given by $\cos\;\theta_{w}^{c}=\gamma_{\mathrm{alc}}/\gamma_{w}$. For this contact angle, the liquid adhesion tension is $\gamma \cos\;\theta =\gamma_{\mathrm{alc}}$ and is independent of the mixture composition, as shown in Fig. 4B by a dashed red line. For contact angles below and above $\theta_{w}^{c}$, adding alcohol has the opposite effect on γcosθ.

With the obtained γcosθ, we can finally compute how the adsorption free energy given by Eq. 1 varies with ethanol/water composition. To that end, we use $w_{\mathrm{ll}}$ and $w_{\mathrm{sl}}$ that we obtained from our simulation model and assume that they remain unaffected by ethanol. By solid lines, we show the prediction up to 20 mol% of ethanol in Fig. 4C. One should note that for higher ethanol fractions, lipids start to dissolve and form micelles in the solution (43), and our two-state model, which accounts only for adsorbed monolayer and bilayer, breaks down. As seen, the oxidized silicon substrate ($\theta_{w}\approx 45^{\circ }$) is resistant to monolayer adsorption in pure water. Ethanol reduces the repulsion, and at around 7 mol% (≈ 20 vol%), the substrate eventually becomes favorable to monolayer adsorption. A similar, albeit much weaker, effect of ethanol is observed for the fresh silicon substrate ($\theta_{w}\approx 66^{\circ }$). Namely, both cases lie below the critical value of $\theta_{w}^{c}=72^{\circ }$, such that ethanol decreases the liquid adhesion tension and, with that, the adsorption free energy. Finally, for the most hydrophobic surface ($\theta_{w}\approx 104^{\circ }$), the effect of ethanol is the opposite because, in this case, $\theta_{w}>\theta_{w}^{c}$: Ethanol now weakens strong adsorption, but not enough to cause desorption.

The overall conclusion drawn from Fig. 4C and the Zisman-derived relation (Eq. 8) is that increasing the ethanol content gradually pushes the liquid adhesion tension toward $\gamma \cos\;\theta \to \gamma_{\mathrm{alc}}$ and with that, the free energy (Eq. 1) approaches


(9) $$\Delta F_{\mathrm{alc}}/A=w_{\mathrm{ll}}/2-w_{\mathrm{sl}}+\gamma_{\mathrm{alc}}$$

whose value is $\Delta F_{\mathrm{alc}}/A\approx -8$ mN/m and is independent of the contact angle (shown by a dashed red line in Fig. 4C). Note again that this limit is hypothetical, as the lipids dissolve at high alcohol concentrations. Nevertheless, the analysis suggests that organic cosolvent leads to weak adsorption states of the monolayer regardless of the contact angle. Namely, increasing the organic component makes the solvent less polar, thereby weakening the hydrophobic effect. In less polar solvents, the contrast between hydrophobic lipid tails and the solvent becomes smaller than in an aqueous solvent. We demonstrated solvent exchange in the case of ethanol/water mixtures. As per the general, albeit approximate, validity of the Zisman relation, we can expect that qualitatively the same behavior applies to other simple organic solvents as well. Since small amounts of organic cosolvents weaken the driving force for adsorption, one can also expect that they slow down the adsorption kinetics. Thus, a well-controlled exchange between the organic and aqueous components may be useful for preparing uniform and defect-free monolayers on hydrophobic surfaces.

## Conclusions

With basic free-energy principles corroborated by molecular simulations, we have analyzed the thermodynamics of lipid monolayer formation on noncharged and nonpolarizable solid surfaces in aqueous solutions and aqueous mixtures with organic solvents. Hydrophilic model substrates with water contact angles below the critical value of $\theta_{\mathrm{ads}}=66^{\circ }$ are resistant against monolayer formation since the bilayer in bulk solution is thermodynamically preferred over the adsorbed monolayer. On the contrary, hydrophobic and weakly hydrophilic substrates that exceed this critical value are susceptible to monolayer adsorption and thus suitable for lipid coating applications. The adsorption angle $\theta_{\mathrm{ads}}$, which designates the onset of monolayer adsorption, turns out to be only weakly dependent on the substrate material and is surprisingly universal in aqueous environments. However, the addition of a less polar solvent to the aqueous medium generally lowers $\theta_{\mathrm{ads}}$ and increases the ability for monolayer formation on hydrophilic surfaces—as long as the layered lipid structures remain stable. Organic solvent–water mixing therefore also represents a viable and simple way to tune the driving force for monolayer adsorption and, consequently, its kinetics.

The introduced theoretical framework and its outcomes provide not only insight into the thermodynamics of lipid monolayer adsorption on atomistically flat substrates but also offer a good starting point to investigate more complex surfaces. Additional layers of complexity may include surface charges, substrate polarizability, surface topography, or curved surfaces, which can modify the current results and are vital in designing lipid coatings and, therefore, worth exploring in the future.

The significance of the adsorption contact angle established here (60${}^{\circ }$$≲\theta_{\mathrm{ads}}≲$ 70${}^{\circ }$) extends beyond lipid monolayer adsorption alone. In a broader context, the same general principles also seem to apply to the adsorption of other (bio-)macromolecular layers to surfaces since layers of adsorbing molecules are typically of an amphiphilic character. In fact, our results appear to rationalize the value of a similar critical contact angle, sometimes referred to as the “Berg limit,” which was reported for the capability of solid surfaces to adsorb biological matter and to be colonized by organisms (51–53) and has more recently been associated also to the attraction-to-repulsion transition of planar surfaces in water (54, 30, 31).

## Methods

### Simulation details

We used the SPC/E (55) water model because of its simplicity and computational efficiency. Lipids and the substrate were modeled with the CHARMM36/LJ-PME force field (33), which is optimized for the Particle-Mesh-Ewald (PME) summation of Lennard-Jones (LJ) interactions. The use of the CHARMM36 force field was motivated by a known good performance for lipid bilayers (56) as well as our systematic analysis in which we analyzed how well various force fields reproduce the surface tension of decane. The analysis (shown Fig. S1) has demonstrated that the CHARMM36/LJ-PME force field yields excellent agreement with experimental values and outperforms other force fields. The simulations were performed with the Gromacs 2022.1 simulation package (32). Electrostatic and LJ interactions were treated using PME methods with a 1.4 nm real-space cutoff. The temperature of 300 K was controlled by the v-rescale thermostat (57) with a time constant of 0.1 ps.

### Substrate model (SAM)

The solid substrate was modeled as a self-assembled monolayer of ten-carbon-atom long alkyl chains terminated by hydroxyl (OH) groups (i.e. decanol molecules). The molecules were arranged in a hexagonal lattice with a nearest-neighbor distance of 0.497 nm, mimicking SAMs on a gold surface, Au(111) (58, 59)—note that the gold surface was not simulated. The molecules were tilted by 30${}^{\circ }$ relative to the normal of the substrate (60). To preserve the arrangement of the molecules, each of them was restrained by harmonic potentials at two positions: The second C-atom from the OH group was restrained with a force constant of $k_{x}=k_{y}=k_{z}=300$ kJ mol${}^{-1}$ nm${}^{-2}$, and the terminal C-atom (in the CH${}_{3}$ group) with a force constant of $k_{x}=k_{y}=k_{z}=500$ kJ mol${}^{-1}$ nm${}^{-2}$. The imposed restraints render the SAM quite rigid and solid-like, as can be seen from the layered nature of its density profile in Fig. S7B. However, the SAM’s rigidity, set by the choice of the restraints, is expected to have little effect on its adhesion properties with liquids, such as water and lipid tails (31).

The interaction potentials of the SAM were described by the CHARMM36/LJ-PME force field to be compatible with lipids. The substrate’s polarity and contact angle were tuned by scaling the partial charges of the hydroxyl group and its three nearest neighbors in the range from 0 to 1. The same philosophy of contact angle control was used before but with a different SAM structure and a different force field (30, 31, 54, 61). Since the current SAM model has not been used before, we had to measure the contact angles for each polarity. This was done with the sessile droplet method, in which cylindrical droplets of different sizes on the substrate were simulated (61). The macroscopic contact angle for each polarity was computed by extrapolating the measured contact angles to infinitely large droplets. See the Supplementary material for details (Figs. S2 and S3 and Table S1).

## Supplementary Material

pgad190_Supplementary_Data

## Data Availability

Simulation files are available at https://dx.doi.org/10.5281/zenodo.7981121. The Python code for computing contact angles from MD trajectories is available at https://dx.doi.org/10.5281/zenodo.7982303.

## References

[1] Ramanathan M *et al*. 2013. Amphiphile nanoarchitectonics: from basic physical chemistry to advanced applications. Phys Chem Chem Phys. 15:10580–10611. 23639971 10.1039/c3cp50620g

[2] Kaganer VM , Möhwald H, Dutta P. 1999. Structure and phase transitions in Langmuir monolayers. Rev Mod Phys. 71:779.

[3] Plant AL . 1999. Supported hybrid bilayer membranes as rugged cell membrane mimics. Langmuir. 15:5128–5135.

[4] Schneck E *et al*. 2009. Calcium ions induce collapse of charged O-side chains of lipopolysaccharides from *Pseudomonas aeruginosa*. J R Soc Interface. 6:S671–S678. 19605401 10.1098/rsif.2009.0190.focus PMC2843973

[5] Sackmann E . 1996. Supported membranes: scientific and practical applications. Science. 271:43–48. 8539599 10.1126/science.271.5245.43

[6] Richter RP , Bérat R, Brisson AR. 2006. Formation of solid-supported lipid bilayers: an integrated view. Langmuir. 22:3497–3505. 16584220 10.1021/la052687c

[7] Castellana ET , Cremer PS. 2006. Solid supported lipid bilayers: from biophysical studies to sensor design. Surf Sci Rep. 61:429–444. 32287559 10.1016/j.surfrep.2006.06.001 PMC7114318

[8] Brockman H . 1999. Lipid monolayers: why use half a membrane to characterize protein-membrane interactions? Curr Opin Struct Biol. 9:438–443. 10449364 10.1016/S0959-440X(99)80061-X

[9] Giner-Casares JJ , Brezesinski G, Möhwald H. 2014. Langmuir monolayers as unique physical models. Curr Opin Colloid Interface Sci. 19:176–182.

[10] Pedrosa M , Maldonado-Valderrama J, Gálvez-Ruiz MJ. 2022. Interactions between curcumin and cell membrane models by Langmuir monolayers. Colloids Surf B: Biointerfaces. 217:112636. 35738079 10.1016/j.colsurfb.2022.112636

[11] Glasmästar K , Larsson C, Höök F, Kasemo B. 2002. Protein adsorption on supported phospholipid bilayers. J Colloid Interface Sci. 246:40–47. 16290382 10.1006/jcis.2001.8060

[12] Follmann HD *et al*. 2012. Antiadhesive and antibacterial multilayer films via layer-by-layer assembly of TMC/heparin complexes. Biomacromolecules. 13:3711–3722. 22998803 10.1021/bm3011962

[13] Persson F *et al*. 2012. Lipid-based passivation in nanofluidics. Nano Lett. 12:2260–2265. 22432814 10.1021/nl204535h PMC3348678

[14] Ma GJ *et al*. 2022. Lipid coating technology: a potential solution to address the problem of sticky containers and vanishing drugs. View. 3:20200078.

[15] Silva JS , De Barros A, Constantino CJ, Simoes FR, Ferreira M. 2014. Layer-by-layer films based on carbon nanotubes and polyaniline for detecting 2-chlorophenol. J Nanosci Nanotechnol. 14:6586–6592. 25924303 10.1166/jnn.2014.9376

[16] Van Schooneveld MM *et al*. 2008. Improved biocompatibility and pharmacokinetics of silica nanoparticles by means of a lipid coating: a multimodality investigation. Nano Lett. 8:2517–2525. 18624389 10.1021/nl801596a

[17] De Villiers MM , Otto DP, Strydom SJ, Lvov YM. 2011. Introduction to nanocoatings produced by layer-by-layer (LbL) self-assembly. Adv Drug Deliv Rev. 63:701–715. 21699936 10.1016/j.addr.2011.05.011

[18] Luchini A , Vitiello G. 2019. Understanding the nano-bio interfaces: lipid-coatings for inorganic nanoparticles as promising strategy for biomedical applications. Front Chem. 7:343. 31165058 10.3389/fchem.2019.00343 PMC6534186

[19] Kuai R , Li D, Chen YE, Moon JJ, Schwendeman A. 2016. High-density lipoproteins: nature’s multifunctional nanoparticles. ACS Nano. 10:3015–3041. 26889958 10.1021/acsnano.5b07522 PMC4918468

[20] Ishihara K *et al*. 1992. Hemocompatibility of human whole blood on polymers with a phospholipid polar group and its mechanism. J Biomed Mater Res. 26:1543–1552. 1484061 10.1002/jbm.820261202

[21] Trojanowicz M . 2001. Miniaturized biochemical sensing devices based on planar bilayer lipid membranes. Fresen J Anal Chem. 371:246–260. 10.1007/s002160101005 11678199

[22] Kochanowski A *et al*. 2011. Examination of the inflammatory response following implantation of titanium plates coated with phospholipids in rats. J Mater Sci: Mater Med. 22:1015–1026. 21455678 10.1007/s10856-011-4287-6

[23] Linseisen FM , Hetzer M, Brumm T, Bayerl TM. 1997. Differences in the physical properties of lipid monolayers and bilayers on a spherical solid support. Biophys J. 72:1659–1667. 9083669 10.1016/S0006-3495(97)78811-8 PMC1184359

[24] Lahiri J , Jonas SJ, Frutos AG, Kalal P, Fang Y. 2001. Lipid microarrays. Biomed Microdevices. 3:157–164.

[25] Lenz P , Ajo-Franklin CM, Boxer SG. 2004. Patterned supported lipid bilayers and monolayers on poly (dimethylsiloxane). Langmuir. 20:11092–11099. 15568862 10.1021/la048450i

[26] Babayco CB *et al*. 2010. A comparison of lateral diffusion in supported lipid monolayers and bilayers. Soft Matter. 6:5877–5881.

[27] Groves JT , Ulman N, Boxer SG. 1997. Micropatterning fluid lipid bilayers on solid supports. Science. 275:651–653. 9005848 10.1126/science.275.5300.651

[28] Mornet S , Lambert O, Duguet E, Brisson A. 2005. The formation of supported lipid bilayers on silica nanoparticles revealed by cryoelectron microscopy. Nano Lett. 5:281–285. 15794611 10.1021/nl048153y

[29] Troutier A-L , Ladavière C. 2007. An overview of lipid membrane supported by colloidal particles. Adv Colloid Interface Sci. 133:1–21. 17397791 10.1016/j.cis.2007.02.003

[30] Kanduč M , Netz RR. 2015. From hydration repulsion to dry adhesion between asymmetric hydrophilic and hydrophobic surfaces. Proc Natl Acad Sci USA. 112:12338–12343. 26392526 10.1073/pnas.1504919112 PMC4603451

[31] Kanduč M , Schlaich A, Schneck E, Netz RR. 2016. Water-mediated interactions between hydrophilic and hydrophobic surfaces. Langmuir. 32:8767–8782. 27487420 10.1021/acs.langmuir.6b01727

[32] van der Spoel D *et al*. 2005. GROMACS: fast, flexible, and free. J Comput Chem. 26:1701–1718. 16211538 10.1002/jcc.20291

[33] Yu Y *et al*. 2021. CHARMM36 lipid force field with explicit treatment of long-range dispersion: parametrization and validation for phosphatidylethanolamine, phosphatidylglycerol, and ether lipids. J Chem Theory Comput. 17:1581–1595. 33620194 10.1021/acs.jctc.0c01327 PMC8130185

[34] Carlson S *et al*. 2021. Hydrophobicity of self-assembled monolayers of alkanes: fluorination, density, roughness, and Lennard-Jones cutoffs. Langmuir. 37:13846–13858. 34787431 10.1021/acs.langmuir.1c02187

[35] Jasper JJ , Kring EV. 1955. The isobaric surface tensions and thermodynamic properties of the surfaces of a series of n-alkanes, C5 to C18, 1-alkenes, C6 to C16, and of n-decylcyclopentane, n-decylcyclohexane and n-decylbenzene. J Phys Chem. 59:1019–1021.

[36] Rego NB , Ferguson AL, Patel AJ. 2022. Learning the relationship between nanoscale chemical patterning and hydrophobicity. Proc Natl Acad Sci USA. 119:e2200018119. 10.1073/pnas.2200018119 PMC9860318 36409904

[37] Basařová P , Váchová T, Bartovská L. 2016. Atypical wetting behaviour of alcohol–water mixtures on hydrophobic surfaces. Colloids Surf A: Physicochem Eng Asp. 489:200–206.

[38] Vega C , MacDowell L, López-Rodríguez A. 1999. Excess properties of mixtures of n-alkanes from perturbation theory. J Chem Phys. 111:3192–3202.

[39] Esteban B , Riba J-R, Baquero G, Rius A, Puig R. 2012. Temperature dependence of density and viscosity of vegetable oils. Biomass Bioenergy. 42:164–171.

[40] Ingram BT . 1974. Wetting of silica by n-alkanes. J Chem Soc Faraday Trans 1. 70:868–876.

[41] Jańczuk B . 1986. Effect of n-alkanes on wettability of graphite. Fuel. 65:113–116.

[42] Hochrein MB , Reich C, Krause B, Rädler JO, Nickel B. 2006. Structure and mobility of lipid membranes on a thermoplastic substrate. Langmuir. 22:538–545. 16401100 10.1021/la051820y

[43] Hohner AO , David MPC, Rädler JO. 2010. Controlled solvent-exchange deposition of phospholipid membranes onto solid surfaces. Biointerphases. 5:1–8. 20408729 10.1116/1.3319326

[44] Ferhan AR *et al*. 2019. Solvent-assisted preparation of supported lipid bilayers. Nat Protoc. 14:2091–2118. 31175346 10.1038/s41596-019-0174-2

[45] Ly HV , Block DE, Longo ML. 2002. Interfacial tension effect of ethanol on lipid bilayer rigidity, stability, and area/molecule: a micropipet aspiration approach. Langmuir. 18:8988–8995.

[46] Ly HV , Longo ML. 2004. The influence of short-chain alcohols on interfacial tension, mechanical properties, area/molecule, and permeability of fluid lipid bilayers. Biophys J. 87:1013–1033. 15298907 10.1529/biophysj.103.034280 PMC1304443

[47] Spencer S , Andrews G, Deacon C. 2013. Contact angle of ethanol–water solutions on crystalline and mesoporous silicon. Semicond Sci Technol. 28:055011.

[48] von Bahr M , Tiberg F, Zhmud BV. 1999. Spreading dynamics of surfactant solutions. Langmuir. 15:7069–7075.

[49] Staniscia F , Guzman HV, Kanduč M. 2022. Tuning contact angles of aqueous droplets on hydrophilic and hydrophobic surfaces by surfactants. J Phys Chem B. 126:3374–3384. 35468298 10.1021/acs.jpcb.2c01599 PMC9082615

[50] Bera B *et al*. 2018. Counteracting interfacial energetics for wetting of hydrophobic surfaces in the presence of surfactants. Langmuir. 34:12344–12349. 30240229 10.1021/acs.langmuir.8b02874 PMC6193251

[51] Vogler EA . 1998. Structure and reactivity of water at biomaterial surfaces. Adv Colloid Interface Sci. 74:69–117. 9561719 10.1016/s0001-8686(97)00040-7

[52] Rosenhahn A , Schilp S, Kreuzer HJ, Grunze M. 2010. The role of “inert” surface chemistry in marine biofouling prevention. Phys Chem Chem Phys. 12:4275–4286. 20407695 10.1039/c001968m

[53] Ishida N , Kinoshita N, Miyahara M, Higashitani K. 1999. Effects of hydrophobizing methods of surfaces on the interaction in aqueous solutions. J Colloid Interface Sci. 216:387–393. 10421746 10.1006/jcis.1999.6329

[54] Kanduč M , Schneck E, Netz RR. 2014. Attraction between hydrated hydrophilic surfaces. Chem Phys Lett. 610–611:375–380.

[55] Berendsen HJC , Grigera JR, Straatsma TP. 1987. The missing term in effective pair potentials. J Phys Chem. 91:6269–6271.

[56] Botan A , *et al*. 2015. Toward atomistic resolution structure of phosphatidylcholine headgroup and glycerol backbone at different ambient conditions. J Phys Chem B. 119:15075–15088. 26509669 10.1021/acs.jpcb.5b04878 PMC4677354

[57] Bussi G , Donadio D, Parrinello M. 2007. Canonical sampling through velocity rescaling. J Chem Phys. 126:014101. 17212484 10.1063/1.2408420

[58] Strong L , Whitesides GM. 1988. Structures of self-assembled monolayer films of organosulfur compounds adsorbed on gold single crystals: electron diffraction studies. Langmuir. 4:546–558.

[59] Chidsey CE , Loiacono DN. 1990. Chemical functionality in self-assembled monolayers: structural and electrochemical properties. Langmuir. 6:682–691.

[60] Fenter P , Eberhardt A, Liang K, Eisenberger P. 1997. Epitaxy and chainlength dependent strain in self-assembled monolayers. J Chem Phys. 106:1600–1608.

[61] Kanduč M . 2017. Going beyond the standard line tension: size-dependent contact angles of water nanodroplets. J Chem Phys. 147:174701. 29117696 10.1063/1.4990741

