# Correction to: Conditions for the stable adsorption of lipid monolayers to solid surfaces

**DOI:** 10.1093/pnasnexus/pgag098

**Published:** 2026-04-13

This is a correction to: Marin Šako, Fabio Staniscia, Emanuel Schneck, Roland R Netz, Matej Kanduc̆, Conditions for the stable adsorption of lipid monolayers to solid surfaces, *PNAS Nexus*, Volume 2, Issue 6, June 2023, pgad190, https://doi.org/10.1093/pnasnexus/pgad190

The author list and affiliations list should read:

“Marin Šako^a,b^, Fabio Staniscia^a^, Emanuel Schneck^c^, Roland R. Netz^d^ and Matej Kanduc̆ ^a,*^

^a^Department of Theoretical Physics, Jožef Stefan Institute, Jamova 39, Ljubljana, 1000, Slovenia

^b^Faculty of Mathematics and Physics, University of Ljubljana, Jadranska 19, 1000 Ljubljana, Slovenia

^c^Department of Physics, Technische Universität Darmstadt, Hochschulstrasse 8, Darmstadt 64289, Hesse, Germany

^d^Fachbereich Physik, Freie Universität Berlin, Berlin 14195, Germany

The emendation is outlined only in this notice to preserve the version of record.

