# Supplementary material for: Conditions for the stable adsorption of lipid monolayers to solid surfaces
Source: PNAS Nexus. 2023 Jun 7;2(6):pgad190. doi: 10.1093/pnasnexus/pgad190 (PMC10299894; doi:10.1093/pnasnexus/pgad190)
Supplement: pgad190_Supplementary_Data [file pgad190_supplementary_data.pdf]

Supplementary material: Conditions for the stable adsorption of lipid monolayers to solid surfaces

Marin Šako,[†] Fabio Staniscia,[†] Emanuel Schneck,[‡] Roland R. Netz,[¶] and Matej Kanduč[*,†]

†Department of Theoretical Physics, Jožef Stefan Institute, Jamova 39, 1000 Ljubljana, Slovenia
‡Department of Physics, Technische Universität Darmstadt, Hochschulstrasse 8, 64289 Darmstadt, Germany
¶Fachbereich Physik, Freie Universität Berlin, 14195 Berlin, Germany

E-mail: matej.kanduc@ijs.si

1. REVISION OF FORCE FIELDS

The surface tension of liquid alkanes serves as a meaningful proxy for the adhesion free energy between lipid tails in a bilayer. Therefore, those force fields that accurately reproduce the surface tension of alkanes are also expected to capture considerably well the work of adhesion between lipid tails. To this end, we performed simulations of a slab of decane (C10) in its vapor at different temperatures with several popular force fields and different cutoff values (r_{LJ}) of the Lennard-Jones (LJ) interactions. We tested the following force fields: OPLS,[1] L-OPLS,[2] GROMOS,[3] Berger et al.,[4] CHARMM36,[5] and CHARMM36/LJ-PME.[6] The latter one is a modified version of CHARMM36 for lipids optimized for the Particle-Mesh Ewald summation of LJ interactions (i.e., $r_{\mathrm{LJ}} = \infty$).
The surface tensions were obtained from the anisotropy of the pressure tensor.[12] The results are shown in Figure S1 and compared with experimental data from Refs. 7–11. It can be seen that the GROMMOS force field with $r_{\mathrm{LJ}} = 1.4$ nm and the CHARMM36/LJ-PME force field[6] yield the closest match to the experimental values.
In addition, a comprehensive examination of classical PC-lipid force fields by Botan et al.,[13] based on comparing structural order parameters from simulations with experimental NMR data, revealed an exceeding performance of CHARMM-based force fields over alternative options. Therefore, based on the reproducibility of alkane surface tension and structural order parameters, we deem the CHARMM36/LJ-PME force field[6] as the most appropriate selection for our objectives.

2. DETERMINING THE CONTACT ANGLES OF THE SAM

The contact angles of the SAM substrates of different polarities were determined with the sessile droplet method in the cylindrical morphology.[14] For each polarity, we simulated 3–4 water droplets of different sizes for 10 ns. The first 0.8 ns of trajectories were discarded to ensure well-equilibrated states for sampling. The density was calculated by binning the atom positions of water onto a square grid. The density contours, smoothened by a Gaussian noise filter, allowed us to identify the Gibbs dividing surface (GDS) of the water–vapor interface of the droplet. A typical density profile with its GDS contour is shown in Figure S2.
In the next step, we fitted a circle to the GDS, excluding the irregular part of the contour lines near the surface. To determine the substrate–water GDS, we performed additional simulations of a ~ 3 nm thick water film on the substrate. Finally, we calculated the microscopic contact angle θ_μ and the base radius a of each droplet from the intersection between the fitted circle and the substrate–water GDS.

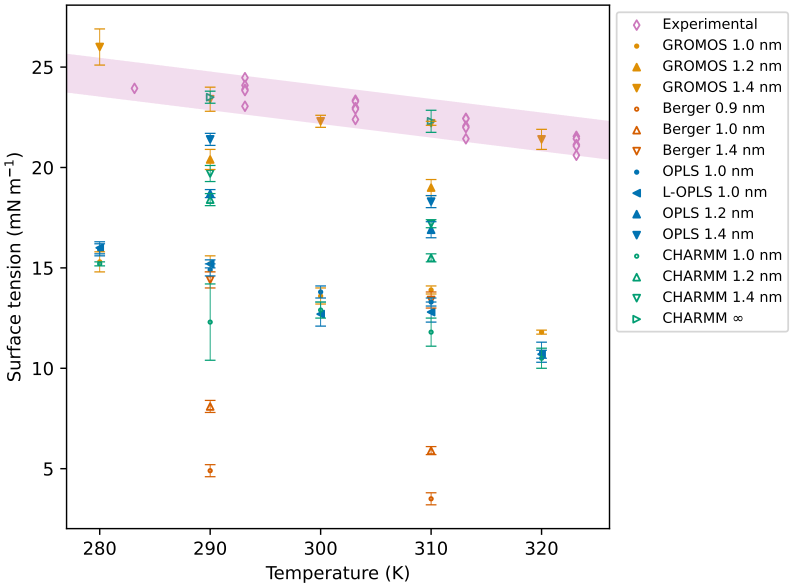

Figure S1: Surface tensions of decane obtained from simulations using different force fields and LJ cutoffs, indicated in the legend. Experimental values from five sources [7–11] are plotted as purple diamonds and highlighted by a shaded stripe. Excellent agreement is obtained with the CHARMM36/LJ-PME ($r_{\mathrm{LJ}} = \infty$).

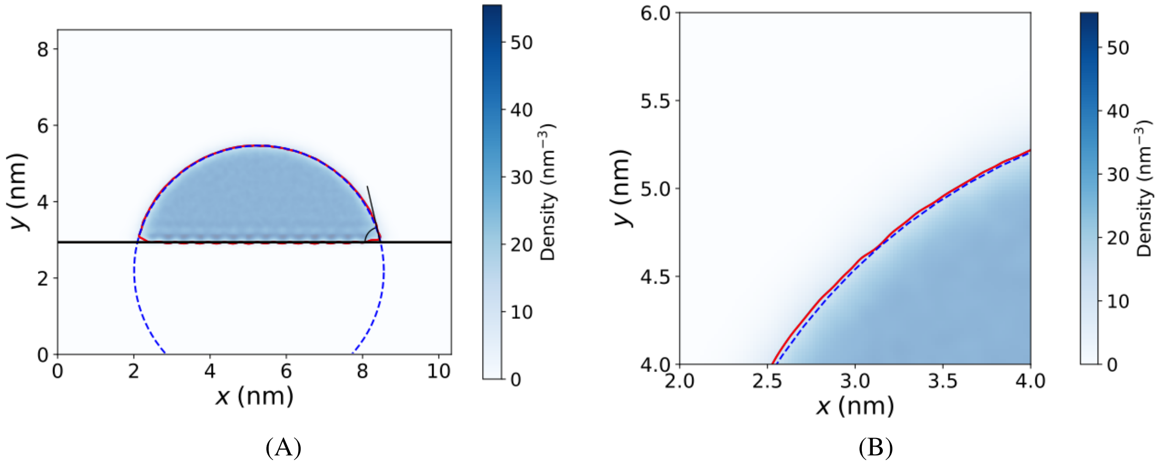

Figure S2: (A) Smoothed density plot of a droplet on a SAM with polarity 0.6 and (B) its zoomed-in area. The continuous red line is the GDS, the dashed blue line is a circular fit of the upper part of the GDS, and the horizontal black line is the location of the substrate. The contact angle is shown at the right intersection between the circle and the substrate.

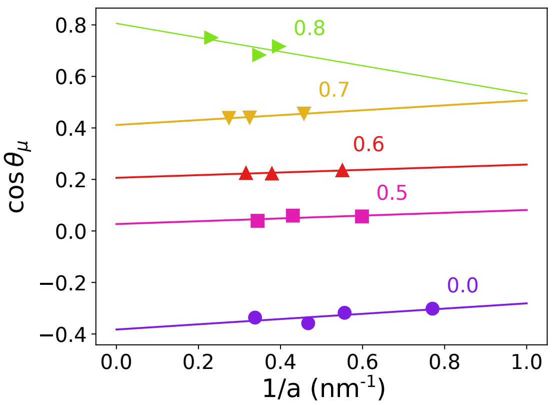

Figure S3: Cosine of the microscopic contact angle vs inverse droplet base radius for different polarities of the substrate (denoted at each set).

Figure S3 shows measured $\cos\theta_\mu$ against the inverse base radius $1/a$ of the droplet for all substrates, except the most polar one (with original OH groups), which exhibits complete wetting ($\theta = 0$). The size scaling can be well described by the modified Young equation,

$$\cos\theta_\mu = \cos\theta + \frac{C}{a} \tag{S1}$$

where C and $\cos\theta$ are fitting parameters. The lines in Figure S3 are the fits of eq S1 to the data points, which allowed us to calculate the macroscopic contact angle θ for infinitely large droplet, $a \to \infty$. The results for macroscopic contact angles are given in Table S1.

Table S1: Macroscopic contact angles obtained from the fits of eq S1 to the MD data points.

Surface polarity	Contact angle θ
0.0	$113° \pm 2°$
0.5	$89° \pm 2°$
0.6	$78° \pm 1°$
0.7	$66° \pm 1°$
0.8	$36° \pm 9°$
1.0	$0°$

3. WORK OF ADHESION

3.1. Lipid–lipid (w_{ll}). The work of adhesion between the bilayer leaflets was computed by separating them apart and integrating the resulting pressure–distance curve. Each leaflet consisted of $N_{\text{lip}} = 25$ DLPC molecules, built with the CHARMM-GUI membrane builder.[15–18] Periodic boundary conditions were applied in all three dimensions. The bilayer was first equilibrated at 1 bar in water, which resulted in lateral box dimensions of 4.0 nm $\times$ 4.0 nm.

In the next step, the bilayer with two water slabs ($\sim$1 nm thick) at each side was placed in a more extended simulation box in the z-direction, with a vapor phase in between (snapshot in Figure S4A). The CH_2 groups in the glycerol part between the lipid tails (see Figure S4B) were positionally restrained in the z-direction with a harmonic potential of spring constant $k_z = 200$ kJ mol^{-1} nm^{-2}. We performed a sequence of NVT simulations (each 2 ns long), whereby the separation between the restraints for both leaflets was increased by 0.05 nm in each succeeding simulation in the sequence. The pressure acting between the leaflets was calculated from the forces in the springs as $F_z = -N_{\text{lip}} k_z (\langle z \rangle - z_0)$, where $\langle z \rangle$ is the time- and ensemble-averaged position of the restrained C-atoms in each leaflet and z_0 is the position of the restraining potential. The uncertainty in the pressure was estimated from the block-averaging method over time.

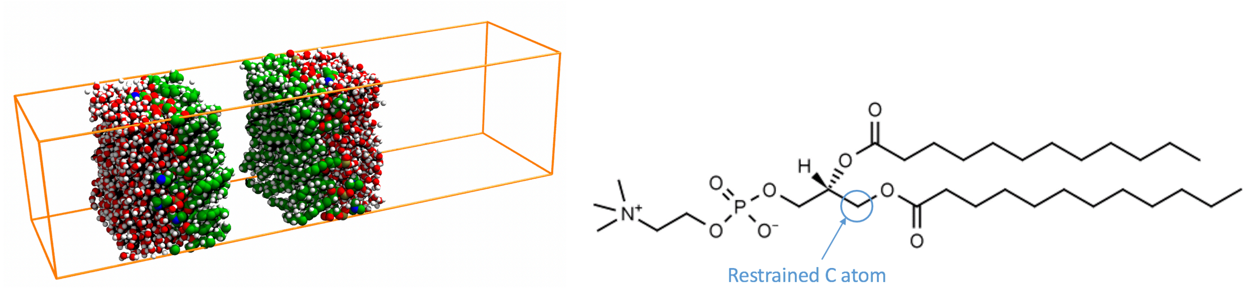

(A) (B)

Figure S4: Restrained C atom in the DLPC molecule.

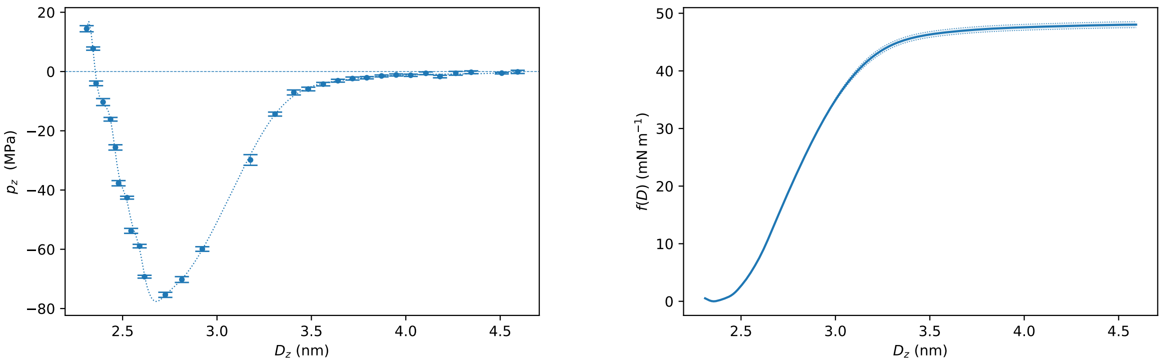

(A) (B)

Figure S5: (A) Pressure between DLPC leaflets as a function of the separation between the restrained C-atoms, D. The dotted line is a fitted spline curve, used for integration. (B) The free energy calculated via eq S2.

The free energy per unit area, $f(D)$, of the leaflets as a function of distance between the restrained C-atom in the right (R) and left (L) leaflet $D = \langle z \rangle_\mathrm{R} - \langle z \rangle_\mathrm{L}$ was obtained by integrating the pressure acting between the leaflets,

$$f(D) = -\int_{D_0}^{D} p_z(D')\mathrm{d}D' \tag{S2}$$

where D_0 is the equilibrium distance of the leaflets at the atmospheric pressure (i.e., $p_z(D_0) = 1$ bar). The numerical integration was performed for interpolated pressure $p_z(D)$, based on cubic splines in the “scipy.interpolate” python package.[19] The uncertainty of the integral was estimated from the error propagation of the trapezoidal integration scheme as

$$\delta f(D) = \left(\sum_i \delta p_{z,i}^2 \Delta D_i^2 \right)^{1/2} \tag{S3}$$

where $\delta p_{z,i}$ is the pressure uncertainty at the i-th separation and $\Delta D_i = D_i - D_{i-1}$ is the difference between the i-th and $(i-1)$th separation. Finally, the work of adhesion is the value of the free energy at an infinite separation $w_\mathrm{ll} = f(D \to \infty)$.

3.2. Effect of ethanol: Stretched bilayers. The main structural effect of ethanol in water is the expansion of the surface area of the lipid bilayer.[20,21] We did not explicitly simulate ethanol but emulated its effect by simulating an expanded (stretched) bilayer in lateral directions. To that end, we scaled the lateral box dimensions by a factor of 1.05 (corresponding to ~ 5 mol% ethanol) and 1.1 (corresponding to ~ 10 mol% ethanol).[20,21] Following the same procedure of pressure integration as for the non-stretched case, we obtained the results summarized in Table S2.

Table S2: Left to right: lateral box sizes, lateral area increase, the work of adhesion, and the overlap integral. The quantities with the subscripts 0 correspond to the non-stretched case.

$L_{x,y}$ (nm)	A/A_0	w_{ll} (mN m^{-1})	S/S_0
4.0	1	49.4 ± 0.6	1
4.2	1.1	51.5 ± 0.6	1.32
4.4	1.21	55.7 ± 0.4	1.61

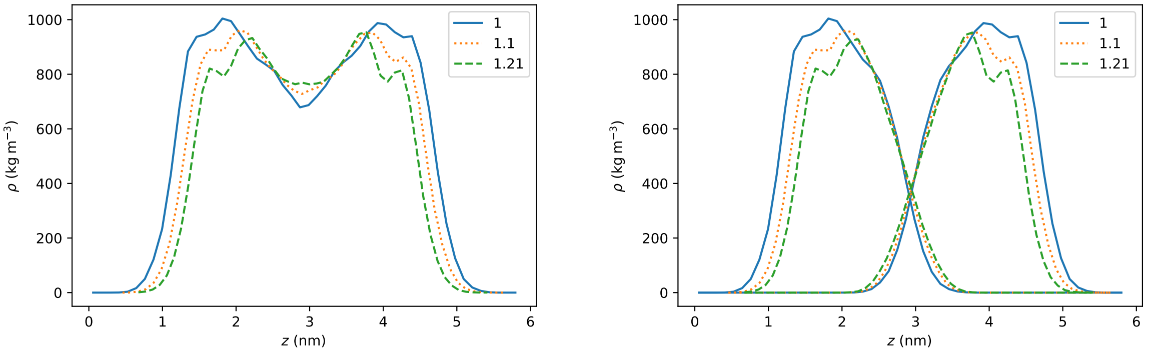

(A) (B)

Figure S6: Density profiles along the z-axis for different area stretching factors of (A) the entire bilayer and (B) decomposed into the left and right leaflets.

Lateral stretching of the bilayer increases the adhesion between the leaflets a little (i.e., by around 10% for the larger extension). At the same time, stretching the bilayer results in its thinning, such that the density of the tail region remains little affected, as seen in Figure S7A. Furthermore, the methyl dip in the middle becomes also less prominent compared to the stretched cases, suggesting that the tails intercalate into the opposing leaflet and make better contacts that increase the adhesion. This presumption is further supported in Figure S7B, in which the bilayer densities are decomposed into contributions from the left and right leaflets, showing that the two leaflet densities overlap more for laterally stretched cases. The overlap of the leaflets can be quantified by the overlap integral, defined as

$$S = \int_{-\infty}^{\infty} \rho_{\text{L}}(z)\rho_{\text{R}}(z)\text{d}z \tag{S4}$$

where $\rho_{\text{L}}(z)$ and $\rho_{\text{R}}(z)$ are the densities of the left and right leaflet, respectively. The overlap integral indeed considerably increases with lateral stretching, as seen from Table S2.

3.3. Substrate–lipid (w_{sl}). For evaluating the work of adhesion between the SAM and the lipid monolayer, we followed the same procedure as for evaluating the leaflet–leaflet work of adhesion, except that one of the monolayer leaflets was replaced by the SAM. Simulations were performed for all six different substrate polarities and three stretching configurations of the lipid monolayer. The number $N_{\text{lip}} = 25$ of lipids was the same in all simulations,

therefore, larger SAM substrates were built to fit the simulation box dimensions for stretched lipid configurations. The lateral simulation box dimensions are given in Table S3.

Table S3: Simulation box dimensions for the SAM/monolayer system and the resulting overlap integral.

L_x (nm)	L_y (nm)	A/A_0	S/S_0
4.0	3.9	1	1
4.5	3.9	1.125	1.09
4.5	4.3	1.24	0.92

The results for w_{sl} are shown in Figure S7A. It can be seen that w_{sl} for the nonpolar case ($\theta = 113°$) nearly coincides with the lipid–lipid work of adhesion, w_{ll}. This is not surprising, as the nonpolar SAM substrate is similar to lipid tails. The adhesion increases by about 10% for the most polar substrate ($\theta = 0°$). There is no noteworthy difference in the work of adhesion between differently stretched monolayers. As opposed to the lipid–lipid case, where stretching resulted in enhanced intercalation of lipid tails, the SAM is impenetrable to lipid tails, and hence intercalation cannot occur (see Figure S7b). Accordingly, the overlap integral of the SAM and lipid densities does not change significantly with stretching, as shown in Table S3.

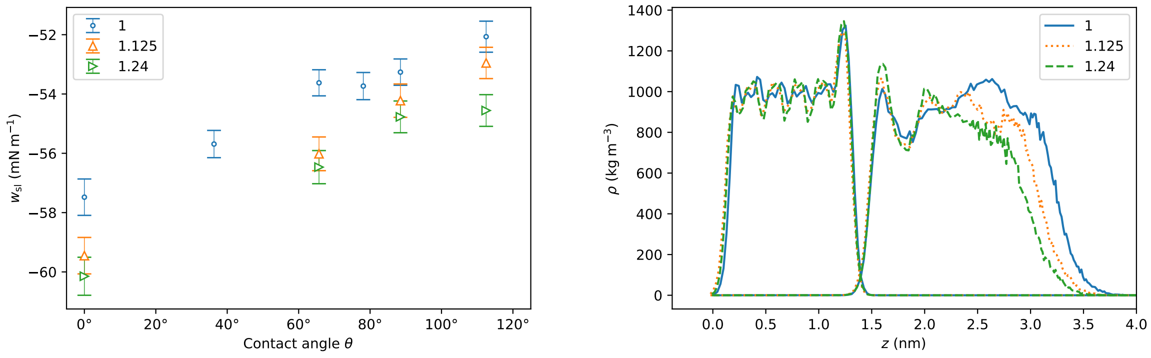

(A) (B)

Figure S7: (A) Work of adhesion between a DLPC monolayer and SAM as a function of its surface contact angle for three different area stretching factors of the monolayer. (B) Density profiles of the SAM (left) and DLPC monolayer (right) for three different area stretching factors of the monolayer.

4. FINITE-SIZE EFFECTS IN AN AQUEOUS MEDIUM

The analysis in the main paper, centered around eq 1, is applicable to sufficiently large lipid layers. However, for smaller layers, additional factors may become significant, and we examine these considerations in the subsequent analysis. To incorporate any additional contributions to the free energy, we can include a correction term $\Delta F_{\text{corr}}/A$ (which will be specified further below) in eq 5,

$$\Delta F/A \approx \gamma(\cos\theta - \cos\theta_{\text{ads}}) + \Delta F_{\text{corr}}/A \tag{S5}$$

In this case, the new equation can be re-expressed in the form of eq 5 by replacing the "macroscopic" adsorption contact angle θ_{ads}, valid for an infinitely large monolayer, by the corrected "microscopic" adsorption contact angle $\theta_{\text{ads}}^{(\text{mic})}$, defined as

$$\cos\theta_{\text{ads}}^{(\text{mic})} = \cos\theta_{\text{ads}} - \frac{\Delta F_{\text{corr}}}{\pi\gamma R^2} \tag{S6}$$

Here, we have assumed that the adsorbed monolayer is a disk of radius R, with the surface area $A = \pi R^2$. In the case when $\Delta F_{\rm corr}$ scales more slowly than R^2, the correction term vanishes for large layers and $\theta_{\rm ads}^{\rm (mic)}$ tends to $\theta_{\rm ads}$.

We describe two topologically distinct forms of a finite-sized bilayer: (i) a flat disk (a bicelle) and (ii) a spherical vesicle. In the case of a bilayer disk of radius $R_{\rm b}$, we have to correct its free energy for the edge contribution, $2\pi R_{\rm b}\lambda_{\rm b}$, where $\lambda_{\rm b} \sim 10$–40 pN is the edge tension of the bilayer.[22,23] A similar edge correction applies to the adsorbed monolayer, $2\pi R\lambda_{\rm m}$, where R and $\lambda_{\rm m}$ stand for the radius and edge tension of the monolayer, respectively. The monolayer edge tension $\lambda_{\rm m}$ has been much less studied than $\lambda_{\rm b}$, and it possibly depends on the contact angle of the substrate. It is reasonable to assume that both tensions are of the same order of magnitude, $\lambda_{\rm m} \sim \lambda_{\rm b}$. From the conservation of the lipid area, it follows that $R_{\rm b} = R/\sqrt{2}$. When a bicelle transforms into an adsorbed monolayer, the free energy correction in eq S5 is

$$\Delta F_{\rm corr}^{\rm (bic)} = 2\pi R(\lambda_{\rm m} - \lambda_{\rm b}/\sqrt{2}) \tag{S7}$$

If the bilayer takes the form of a spherical vesicle, the structural properties are influenced by the bending energy penalty instead of edge effects, which can be quantified as $4\pi(2\kappa_{\rm b} + \kappa_{\rm G})$.[24] Here, $\kappa_{\rm b}$ and $\kappa_{\rm G}$ are the bending rigidity and the Gaussian bending rigidity, respectively. For PC bilayers the experimental values are $\kappa_{\rm b} = 10$–$20 k_{\rm B}T$.[24] For $\kappa_{\rm G}$, few studies exist, owing to its difficult measurement, but they suggest $\kappa_{\rm G}$ to be typically several $k_{\rm B}T$ in size and negative.[25] The transition from a vesicle to an adsorbed monolayer has the following correction

$$\Delta F_{\rm corr}^{\rm (ves)} = -4\pi(2\kappa_{\rm b} + \kappa_{\rm G}) + 2\pi R\lambda_{\rm m} \tag{S8}$$

It should be noted that for smaller bilayers, bicelles are thermodynamically preferred over vesicles, and the other way round for larger bilayers. This behavior arises from the competition between bending and edge energies. The size for the spontaneous bicelle-to-vesicle transition can be computed from $\Delta F_{\rm corr}^{\rm (bic)} = \Delta F_{\rm corr}^{\rm (ves)}$, which leads to the critical monolayer radius $R^* = 2\sqrt{2}(2\kappa_{\rm b} + \kappa_{\rm G})/\lambda_{\rm b}$. Choosing $2\kappa_{\rm b} + \kappa_{\rm G} = 20\,k_{\rm B}T$ and $\lambda_{\rm b} = 20$ pN as typical values, gives $R^* = 12$ nm, which is roughly consistent with earlier estimates and experimental observations.[26]

In Figure S8, we plot the corrected, microscopic adsorption contact angle $\theta_{\rm ads}^{\rm (mic)}$ for both topologies as a function of monolayer radius, where we have used three different values for $\lambda_{\rm m}$, as indicated on the graph. At $R = R^*$ the curves for the bicelle and vesicle of the same $\lambda_{\rm m}$ (shown by the same color) intersect. The correction can have either a positive or negative value, but it generally remains within a few degrees. Note that for nanometer-sized monolayers, the continuum description starts to lose accuracy. We can conclude that finite-size corrections become important once the monolayer radius becomes smaller than around 10 nm.

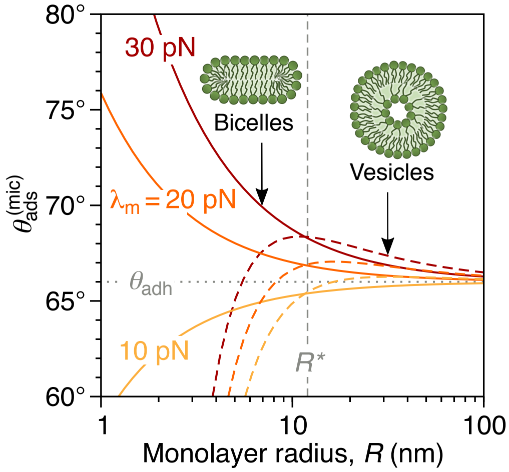

Figure S8: Corrected (microscopic) adsorption contact angle (eq S6) in water for adsorption of a finite monolayer disk of radius R from a preexisting cylindrical bicelle (solid lines, eq S7) or a spherical vesicle (dashed lines, eq S8). The parameters are: $2\kappa_{\rm b} + \kappa_{\rm G} = 20\,k_{\rm B}T$, $\lambda_{\rm b} = 20$ pN, and $\theta_{\rm ads} = 66^\circ$. Three different values for the monolayer edge tension $\lambda_{\rm m}$ are used for both cases as noted in the plot.

Finally, a bilayer can weakly adhere to the solid surface via van der Waals forces, as discussed at the beginning of the main part of the paper. Since the bilayer is a highly hydrophilic surface, hydration repulsion comes into play,[27] causing the bilayer to be separated from the substrate by a thin water film of thickness D_0. The van der Waals correction in this case is[28]

$$\Delta F_{\mathrm{corr}}^{(\mathrm{vdW})}/A = -\frac{A_{\mathrm{H}}}{12\pi D_0^2} \tag{S9}$$

Typical equilibrium distances are $D_0 = 0.3$–3 nm, where the lower limit corresponds to a bilayer comparatively strongly adsorbed to silicon oxide[29,30] and the upper limit to a bilayer very weakly adsorbed to another bilayer.[31] With the Hamaker constant $A_{\mathrm{H}} \approx 1\,k_{\mathrm{B}}T$ of hydrocarbons in water,[28] this range yields $\Delta F_{\mathrm{corr}}/A \approx 0.01$–1 mN/m, which is 2–4 orders of magnitude smaller than the adsorption free energies of the monolayer (Figure 3). Thus, the possible adhesion of the bilayer poses a negligible correction to the adsorption contact angle and justifies our treatment, which does not distinguish between a bilayer in bulk and being adhered to the substrate.

5. SURFACE TENSION AND WATER ADHESION TENSION FROM PUBLISHED DATA

5.1. Surface tension of ethanol/water solution. The surface tension of ethanol/water solution versus ethanol mole fraction (x) from Basařová et al.[32] is shown as circles in Figure S9A. We used the following fitting function

$$\gamma(x) = \gamma_{\mathrm{w}} + c_1 x + c_2(\mathrm{e}^{-\lambda x} - 1) \tag{S10}$$

where $\gamma_{\mathrm{w}} = 72$ mN/m is the surface tension of neat water, and c_1, c_2, and λ are fitting parameters. The fit is shown as a solid line.

5.2. Contact angles of ethanol/water mixtures. We use published experimental data of contact angles of water/ethanol droplets from Spencer et al.[33] and Basařová et al.[32] and plot them as the cosine of the contact angle against the ethanol mole fraction in Figure S9B. We fit these sets with the following functions: a third-order polynomial (both silicon substrates) and a second-order polynomial (silanized glass). The data point for unoxidized silicon with neat water (the square symbol at $x = 0$) is a distinct outlier, which we discard from the fit. Namely, that data point eludes the otherwise smooth trends in the plot, and additionally, it also clearly steps out from the Zisman-based relation in Figure 4B in the main text. Therefore, there is a good reason to doubt its measurement accuracy. The fits are shown as solid lines in Figure S9B.

References

(1) Jorgensen, W. L.; Tirado-Rives, J. The OPLS [Optimized Potentials for Liquid Simulations] Potential Functions for Proteins, Energy Minimizations for Crystals of Cyclic Peptides and Crambin. *J. Am. Chem. Soc.* **1988**, *110*, 1657–1666.

(2) Siu, S. W.; Pluhackova, K.; Böckmann, R. A. Optimization of the OPLS-AA force field for long hydrocarbons. *J. Chem. Theory Comput* **2012**, *8*, 1459–1470.

(3) Oostenbrink, C.; Villa, A.; Mark, A. E.; van Gunsteren, W. F. A biomolecular force field based on the free enthalpy of hydration and solvation: The GROMOS force-field parameter sets 53A5 and 53A6. *J. Comput. Chem.* **2004**, *25*, 1656–1676.

(4) Berger, O.; Edholm, O.; Jähnig, F. Molecular dynamics simulations of a fluid bilayer of dipalmitoylphosphatidylcholine at full hydration, constant pressure, and constant temperature. *Biophys. J.* **1997**, *72*, 2002–2013.

(5) Klauda, J. B.; Venable, R. M.; Freites, J. A.; O'Connor, J. W.; Tobias, D. J.; Mondragon-Ramirez, C.; Vorobyov, I.; MacKerell Jr, A. D.; Pastor, R. W. Update of the CHARMM all-atom additive force field for lipids: validation on six lipid types. *J. Phys. Chem. B* **2010**, *114*, 7830–7843.

(6) Yu, Y.; Kramer, A.; Venable, R. M.; Brooks, B. R.; Klauda, J. B.; Pastor, R. W. CHARMM36 lipid force field with explicit treatment of long-range dispersion: parametrization and validation for phosphatidylethanolamine, phosphatidylglycerol, and ether lipids. *J. Chem. Theory Comput* **2021**, *17*, 1581–1595.

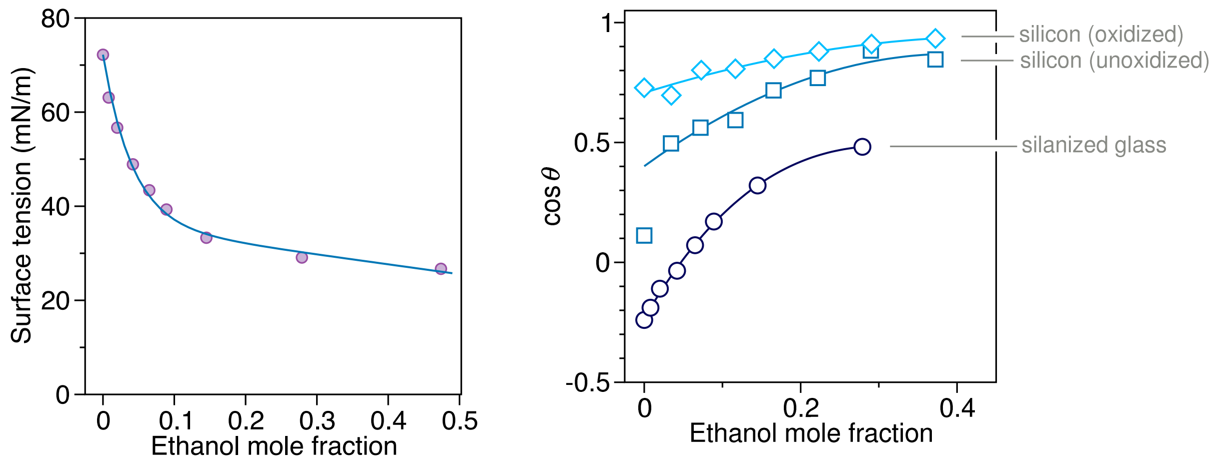

(A) (B)

Figure S9: (A) Surface tension of ethanol/water mixtures from Basařová et al.[32] (circles) and the fit of eq S10 (solid line). (B) Cosine of measured droplet contact angles as a function of ethanol mole fraction on three different substrates: oxidized and unoxidized silicon[33] and silanized glass.[32] Lines are the fits to the data points. We discarded the outlier point at zero ethanol for the unoxidized silicone from the fit.

(7) Jasper, J. J.; Kerr, E. R.; Gregorich, F. The Orthobaric Surface Tensions and Thermodynamic Properties of the Liquid Surfaces of the n—Alkanes, C5 to C28. *J. Am. Chem. Soc.* **1953**, *75*, 5252–5254.

(8) Jasper, J. J. The surface tension of pure liquid compounds. *J. Phys. Chem. Ref. Data* **1972**, *1*, 841–1010.

(9) Queimada, A. J.; Caco, A. I.; Marrucho, I. M.; Coutinho, J. A. Surface tension of decane binary and ternary mixtures with eicosane, docosane, and tetracosane. *J. Chem. Eng. Data* **2005**, *50*, 1043–1046.

(10) Klein, T.; Yan, S.; Cui, J.; Magee, J. W.; Kroenlein, K.; Rausch, M. H.; Koller, T. M.; Fröba, A. P. Liquid viscosity and surface tension of n-hexane, n-octane, n-decane, and n-hexadecane up to 573 K by surface light scattering. *J. Chem. Eng. Data* **2019**, *64*, 4116–4131.

(11) Rolo, L. I.; Caco, A. I.; Queimada, A. J.; Marrucho, I. M.; Coutinho, J. A. Surface tension of heptane, decane, hexadecane, eicosane, and some of their binary mixtures. *J. Chem. Eng. Data* **2002**, *47*, 1442–1445.

(12) Kirkwood, J. G.; Buff, F. P. The statistical mechanical theory of surface tension. *J. Chem. Phys.* **1949**, *17*, 338–343.

(13) Botan, A. et al. Toward atomistic resolution structure of phosphatidylcholine headgroup and glycerol backbone at different ambient conditions. *J. Phys. Chem. B* **2015**, *119*, 15075–15088.

(14) Scocchi, G.; Sergi, D.; D'Angelo, C.; Ortona, A. Wetting and contact-line effects for spherical and cylindrical droplets on graphene layers: A comparative molecular-dynamics investigation. *Phys. Rev. E* **2011**, *84*, 061602.

(15) Jo, S.; Kim, T.; Iyer, V. G.; Im, W. CHARMM-GUI: a web-based graphical user interface for CHARMM. *J. Comput. Chem* **2008**, *29*, 1859–1865.

(16) Brooks, B. R.; Brooks III, C. L.; Mackerell Jr, A. D.; Nilsson, L.; Petrella, R. J.; Roux, B.; Won, Y.; Archontis, G.; Bartels, C.; Boresch, S.; others, o. CHARMM: the biomolecular simulation program. *J. Comput. Chem* **2009**, *30*, 1545–1614.

(17) Lee, J.; Cheng, X.; Swails, J. M.; Yeom, M. S.; Eastman, P. K.; Lemkul, J. A.; Wei, S.; Buckner, J.; Jeong, J. C.; Qi, Y.; others, o. CHARMM-GUI input generator for NAMD, GROMACS, AMBER, OpenMM, and CHARMM/OpenMM simulations using the CHARMM36 additive force field. *J. Chem. Theory Comput.* **2016**, *12*, 405–413.

(18) Wu, E. L.; Cheng, X.; Jo, S.; Rui, H.; Song, K. C.; Dávila-Contreras, E. M.; Qi, Y.; Lee, J.; Monje-Galvan, V.;

Venable, R. M.; others, o. CHARMM-GUI membrane builder toward realistic biological membrane simulations. 2014.

(19) scipy.interpolate python package. https://docs.scipy.org/doc/scipy/tutorial/interpolate.html.

(20) Ly, H. V.; Block, D. E.; Longo, M. L. Interfacial tension effect of ethanol on lipid bilayer rigidity, stability, and area/molecule: a micropipet aspiration approach. *Langmuir* **2002**, *18*, 8988–8995.

(21) Ly, H. V.; Longo, M. L. The influence of short-chain alcohols on interfacial tension, mechanical properties, area/molecule, and permeability of fluid lipid bilayers. *Biophys. J.* **2004**, *87*, 1013–1033.

(22) Portet, T.; Dimova, R. A new method for measuring edge tensions and stability of lipid bilayers: effect of membrane composition. *Biophys. J* **2010**, *99*, 3264–3273.

(23) Dixit, M.; Lazaridis, T. Free energy of hydrophilic and hydrophobic pores in lipid bilayers by free energy perturbation of a restraint. *J. Chem. Phys.* **2020**, *153*, 054101.

(24) Boal, D. *Mechanics of the cell*, edition 1 ed.; Cambridge University Press: The Pitt Building, Trumpington Street, Cambridge, CB2 1RP, UK, 2001.

(25) Claessens, M.; Van Oort, B.; Leermakers, F.; Hoekstra, F.; Stuart, M. C. Charged lipid vesicles: effects of salts on bending rigidity, stability, and size. *Biophys. J.* **2004**, *87*, 3882–3893.

(26) Huang, C.; Quinn, D.; Sadovsky, Y.; Suresh, S.; Hsia, K. J. Formation and size distribution of self-assembled vesicles. *Proceedings of the National Academy of Sciences* **2017**, *114*, 2910–2915.

(27) Kanduč, M.; Schlaich, A.; Schneck, E.; Netz, R. R. Water-Mediated Interactions between Hydrophilic and Hydrophobic Surfaces. *Langmuir* **2016**, *32*, 8767–8782.

(28) Parsegian, V. A. *Van der Waals Forces: A Handbook for Biologists, Chemists, Engineers, and Physicists*; Cambridge University Press, 2005.

(29) Vacklin, H. P.; Tiberg, F.; Fragneto, G.; Thomas, R. K. Composition of supported model membranes determined by neutron reflection. *Langmuir* **2005**, *21*, 2827–2837.

(30) Rondelli, V.; Del Favero, E.; Brocca, P.; Fragneto, G.; Trapp, M.; Mauri, L.; Ciampa, M.; Romani, G.; Braun, C.; Winterstein, L., et al. Directional K+ channel insertion in a single phospholipid bilayer: Neutron reflectometry and electrophysiology in the joint exploration of a model membrane functional platform. *Biochimica et Biophysica Acta (BBA)-General Subjects* **2018**, *1862*, 1742–1750.

(31) Fragneto, G.; Charitat, T.; Daillant, J. Floating lipid bilayers: models for physics and biology. *European Biophysics Journal* **2012**, *41*, 863–874.

(32) Basařová, P.; Váchová, T.; Bartovská, L. Atypical wetting behaviour of alcohol–water mixtures on hydrophobic surfaces. *Colloids Surf. A: Physicochem. Eng. Asp.* **2016**, *489*, 200–206.

(33) Spencer, S.; Andrews, G.; Deacon, C. Contact angle of ethanol–water solutions on crystalline and mesoporous silicon. *Semicond. Sci. Technol.* **2013**, *28*, 055011.
